# Genetic diversity of bovine coronaviruses from recurrent outbreaks on a large-scale cattle farm

**DOI:** 10.1007/s11250-026-05112-8

**Published:** 2026-06-04

**Authors:** Selda Duran-Yelken, Ilke Karayel-Hacioglu, Zelfinaz Aydin, Feray Alkan

**Affiliations:** 1 Department of Virology, Faculty of Veterinary Medicine, Kastamonu University, Kastamonu, Türkiye; 2 Department of Virology, Faculty of Veterinary Medicine, Ankara University, Ankara, Türkiye; 3 Department of Virology, Graduate School of Health Sciences, Ankara University, Ankara, Türkiye; 4 Faculty of Veterinary Medicine, Kastamonu University, Kastamonu, Türkiye

**Keywords:** Bovine coronavirus, Genetic diversity, Molecular analysis, Cattle, Türkiye

## Abstract

This study aimed to characterize the molecular features of bovine coronavirus (BCoV) outbreaks detected on a large-scale cattle farm in Türkiye during three respiratory outbreaks (January, May, and August 2024). Respiratory outbreaks were recorded in all three periods, with the August outbreak also being associated with enteric infection. A total of 52 samples (42 nasal samples and 10 fecal samples) were analyzed using RT-nested PCR targeting the N gene, and 13 samples (12 nasal samples and one fecal sample) tested positive. Among six partial N gene sequences that could be obtained from the outbreak in January (*n* = 1) and August (*n* = 5), two sequences from the August outbreak exhibited a three–amino acid deletion in the RS/SR domain. Furthermore, partial S gene sequences from two samples from the January and August outbreaks revealed different amino acid substitutions. The observed genetic variations indicate the presence of genetically distinct BCoV strains detected during different outbreak events on the farm and support the need for sustained molecular surveillance in cattle farming systems.

**Supplementary Information:**

The online version contains supplementary material available at 10.1007/s11250-026-05112-8.

## Introduction

Bovine coronavirus (BCoV) is a pneumoenteric virus belonging to the family *Coronaviridae*, subgenus *Embecovirus*, species Betacoronavirus 1 (also known as *Betacoronavirus gravedinis*) of the *Betacoronavirus* genus (Fehr and Perlman 2015). Clinical signs of BCoV infection in cattle may include fever, respiratory distress, frequent coughing, nasal discharge, diarrhea, dehydration, and loss of appetite (Socha et al. 2022; Vlasova and Saif 2021). There are conflicting reports on whether differences in the clinical presentation of bovine coronavirus infections are associated with amino acid variations in the N and S proteins and, if so, whether a clear causal relationship exists between specific substitutions and disease phenotype (Cho et al. 2001; Li 2016; Schultze et al. 1991; Hasoksuz et al. 2005; Jeong et al. 2005; Sevinc-Temizkan and Alkan 2021; Suzuki et al. 2020).

The N protein is involved in several key processes such as viral pathogenesis, transcription, and replication. Because it is highly conserved and abundantly expressed during replication, RT-PCR assays targeting the BCoV N gene are commonly used for detecting the virus in clinical samples (Alkan et al. 2011; Lojkić et al. 2015; Mira Fernandes et al. 2018; Keha et al. 2019). The spike (S) protein is encoded by the S gene and serves two primary functions: first, utilizing its N-terminal signal sequence to enter the host cell’s endoplasmic reticulum; second, facilitating the virus’s attachment to the host cell. In the genome, a protease cleavage site located between amino acids 768 and 769 separates the S protein into its S1 and S2 subunits (Takiuchi et al. 2006; Saif 2010). The S1 subunit contains the receptor-binding domain, which facilitates the attachment of the virus to host cell receptors, while the S2 subunit forms a stalk-like structure that mediates the fusion of viral and cellular membranes—a critical step for viral entry into the host cell (Li 2016).

In Türkiye, several studies have reported coronavirus as one of the etiological agents of both respiratory (Hasoksuz et al. 2005; Timurkan et al. 2015; Sevinc-Temizkan and Alkan 2021) and enteric infections (Alkan et al. 2003, 2011; Hasoksuz et al. 2005). In addition, there are reports providing sequence data for specific gene regions or full genomes of coronaviruses detected from different organ systems (Sevinc-Temizkan and Alkan 2021 ; Aksoy et al. 2024; Yilmaz et al. 2024). However, data on the molecular characteristics of coronaviruses circulating in cattle under intensive production systems remain limited, highlighting the need for further epidemiological and genetic investigations.

This study aimed to (i) describe the respiratory outbreak context and longitudinal observations on a large-scale dairy farm in Türkiye in 2024 (January, May, and August), and (ii) perform molecular analyses of BCoV detected during three respiratory outbreaks—the last one of which was also associated with a diarrhoeal outbreak—using partial N and S gene sequencing.

## Materials and methods

### Study background and diagnostic materials

In this study, retrospective nasal samples collected during respiratory outbreaks in January and May in a farm were complemented by additional nasal and faecal sampling at the same farm in August following a new outbreak. Sampling during each outbreak targeted clinically affected animals present at the time for viral aetiological investigation (including BCoV, BRSV, PI3, bovine adenovirus, and BHV-1 in nasal samples, and bovine rotavirus in faecal samples).

Since sampling was performed at a single time point for each period (January, May, and August 2024) and animals were selected based on clinical signs, only one type of sample (nasal swab or fecal) is available per animal. Sampling during each respiratory outbreak focused on clinically affected animals present at the time of sampling rather than being conducted as part of a structured epidemiological survey. Consequently, detailed epidemiological information, including the total number of animals at risk, outbreak duration, and mortality rates, was not consistently available.

The sampled animals were from a farm with a herd size of 2000 located in Kastamonu Province, Türkiye. The herd consisted predominantly of Holstein cattle and was managed under a semi-open housing system commonly used in intensive dairy production. According to information obtained from the farm managers, the most recent introduction of new animals into the herd occurred in 2023 as part of routine herd management. Calves were housed in separate calf pens within the farm facility. As part of the herd health management program, pregnant cows were routinely vaccinated against bovine rotavirus and bovine coronavirus. Respiratory outbreaks occurred on this farm during three different periods: January, May, and August 2024. In January and May, clinical signs were limited to respiratory disease, and the affected calves were aged between 3 and 4 months. In contrast, the August outbreak involved calves aged 0 to 3 months and was associated with both respiratory and enteric infections. In total, 52 samples (10 fecal and 42 nasal swab samples) were tested for BCoV using RT-nested PCR targeting the N gene. The number of samples collected at each sampling time is as follows: A total of 15 and 12 nasal swab samples were collected in January and May, respectively, and 15 nasal swab samples, along with 10 fecal samples, were collected in August.

Diagnostic materials were provided by an authorized veterinarian with the farm owner’s consent. All samples were transported to the laboratory under cold chain and stored at -80 °C until further analysis. All materials used in the study were also tested for other pathogens mentioned as part of diagnostic procedures, and the methodology for the diagnosis and molecular analysis of BCoVs in the scope of this study is detailed in the relevant section.

The collection of samples used in this study was approved by the Ethics Committee of Kastamonu University (Decision No: 15).

### Viral RNA extraction and RT-nested PCR for the N gene

Nasal swab samples were centrifuged, and nucleic acid extraction was performed from the supernatant using the phenol/chloroform method (Sambrook and Russell 2001). Fecal samples were diluted at a 1:10 ratio and centrifuged, and nucleic acid was extracted using TRIzol^®^ LS Reagent (Thermo Fisher Scientific, 10296-028). Following extraction, reverse transcription was performed using a RevertAid first-strand cDNA synthesis kit (Thermo Fisher Scientific). After obtaining cDNA, nested PCR assays were conducted using primers targeting the N gene for virus detection. The RT-nested PCR yielded a 730 bp product in the first round and a 407 bp product in the second round (Cho et al. 2001). Nested-PCR targeting the N gene was carried out using Thermo Scientific DreamTaq DNA Polymerase (#EP0702). PCR conditions were as follows: initial denaturation at 95 °C for 3 min; denaturation at 95 °C for 30 s; annealing at 50 °C for the first round and 58 °C for the second round for 30 s; extension at 72 °C for 1 min; and final extension at 72 °C for 10 min, for a total of 35 cycles.

### RT-PCRs for the S gene

Samples that tested positive for the N gene were subjected to RT-PCR assays targeting the S gene region using nine overlapping primer pairs. These primers were designed to amplify the full S gene (S1 and S2 subunits) (Brandão et al. 2006; Hasoksuz et al. 2002; Martínez et al. 2012) (Table S1). Thermo Scientific DreamTaq DNA Polymerase (#EP0702) was also used in these PCR reactions. PCR conditions were as follows: initial denaturation at 95 °C for 3 min; denaturation at 95 °C for 30 s; annealing temperatures were 51 °C for S-A, 58 °C for S-C and S-H, and 55 °C for the other primer pairs, for 30 s; extension at 72 °C for 1 min; and final extension at 72 °C for 10 min, for a total of 35 cycles.

The amplification products for all RT-PCRs were electrophoresed on 1% agarose gel, stained with SafeView Classic (ABM), and visualized under UV light. The expected-sized amplicons were sequenced in both directions with the same primers used for the amplification, via commercial sequencing services.

### Sequence and phylogenetic analyses

Sequences were aligned with reference virus sequences retrieved from GenBank using the AliView software (Larsson 2014). Phylogenetic analyses of the obtained local strains and other coronavirus sequences from GenBank were conducted using the MEGA X software (Kumar et al. 2018). Maximum likelihood phylogenetic trees were constructed based on the best-fit models selected according to Akaike Information Criterion (AIC) and Bayesian Information Criterion (BIC) values evaluated through model testing. The N gene sequence analyses were performed by the maximum-likelihood method with the Kimura 2-parameter model with a gamma distribution at the nucleotide level, with bootstrap analysis (1,000 replicates) and the p-distance parameter. Phylogenetic analysis of the partial S gene sequences and other coronavirus sequences from GenBank was performed using the Tamura–Nei model with a gamma distribution and a proportion of invariant sites (TN93 + G + I) with a bootstrap value of 1,000 replicates and the p-distance parameter. Additionally, an amino acid alignment was performed at the protein level with the MEGA X software. Reference strains were selected to represent major geographic regions and known BCoV genotypes available in GenBank.

## Results

### BCoV N gene RT-nested PCR and phylogenetic analysis

Out of 52 samples, including 42 nasal and 10 fecal samples, 12 nasal samples tested positive for BCoV—one from January, four from May, and seven from August. These results demonstrate the presence of BCoV throughout all three outbreak periods, despite no evidence of other respiratory viruses being detected during viral etiological investigations targeting BRSV, PI3, bovine adenovirus, and BHV-1 in nasal samples. Additionally, one fecal sample collected in August was also positive for BCoV (Table 1).

**Table 1:** Details of the samples tested and results

Sampling Time	Sample Type	Total Samples	BCoV (+)	Name of strain	Accession no			
					N Gene (1st Round)	N Gene (2nd Round)		S Gene
January	Nasal swab	15	1	BCoV/S2	PV975811		-	PV975817
May	Nasal swab	12	4	-	-		-	-
August	Nasal swab	15	7	BCoV/3243 BCoV/3246 BCoV/3330 BCoV/3185	PV975813 PV975812 - -		- - PV975814 PV975815	- PV975818 - -
	Feces	10	1	BCoV/A1	-		PV975816	-

Sequences could be successfully obtained from six samples— only three from the RT-PCR and three from the subsequent RT-nested PCR, all originating from the January and August sampling periods (Table 1). Unfortunately, some amplification products were faint and did not yield sequences of sufficient quality for sequencing analysis. The sequences of BCoV strains obtained in this study were deposited in GenBank under the following accession numbers: BCoV/S2 (PV975811), BCoV/3246 (PV975812), BCoV/3243 (PV975813), BCoV/3330 (PV975814), BCoV/3185 (PV975815), and BCoV/A1 (PV975816) (Table 1).

**Fig. 1:**
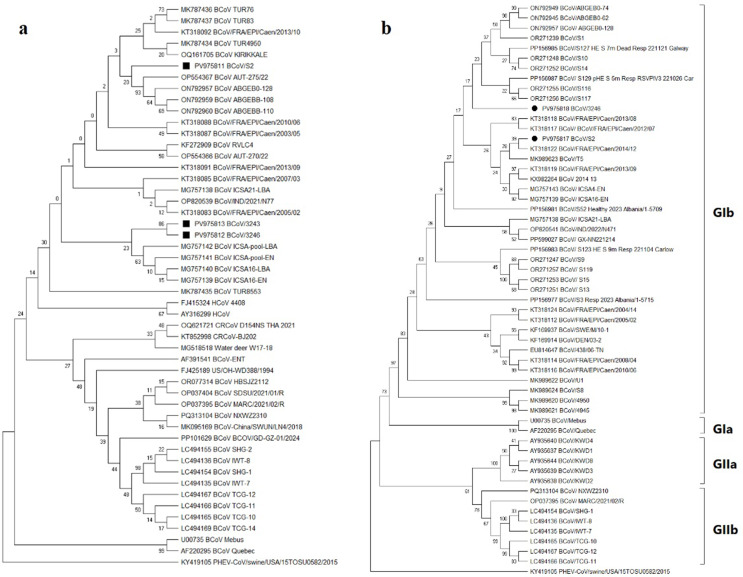
(**a**) Phylogenetic tree illustrating the relationships among bovine coronavirus (BCoV) strains based on the nucleocapsid (N) gene sequences. The tree was constructed using the Kimura 2-parameter model with a gamma distribution. (**b**) Phylogenetic tree illustrating the relationships among BCoV strains based on the spike (S) gene sequences. The tree was constructed using the TN93 + G + I model. Bootstrap values are indicated at the nodes. Sequences obtained in this study are marked with a black square (■) in (**a**) and a black circle (●) in (**b**)

The phylogenetic tree was constructed using the first-round PCR sequences of three samples—BCoV/S2, BCoV/3243, and BCoV/3246—with corresponding BCoV sequences retrieved from GenBank. The BCoV/S2 strain from January clustered with previously reported BCoV strains from Türkiye, whereas BCoV/3243 and BCoV/3246 from August formed a separate branch (Fig. 1a). Comparative analysis of the nucleotide sequences revealed that BCoV/3243 and BCoV/3246 were 100% identical to each other and shared 97.39% similarity with BCoV/S2. The nucleotide similarities between the sequences of the viruses included in the phylogenetic tree and those identified in this study ranged from 95.91% to 99.62% for the BCoV/S2 strain and from 96.65 to 99.62% for the viruses collected in August. Besides, the nucleotide similarities of the sequences obtained in January (BCoV/S2) and August (BCoV/3243 and BCoV/3246) with the Mebus reference strain were estimated as 98.32%, and 96.84%, respectively. At amino acid level, BCoV/S2 was more closely related to the Mebus reference strain (99.44%) than BCoV/3243 and BCoV/3246 (98.32%).

Based on the sequences obtained after the second round of RT-PCR, the six BCoV N gene sequences analyzed in this study exhibited high conservation, with nucleotide and amino acid similarities ranging from 98.58% to 100% and from 99.02% to 100%, respectively. The sequence similarities were calculated based on the overlapping regions shared by sequences.

**Fig. 2:**
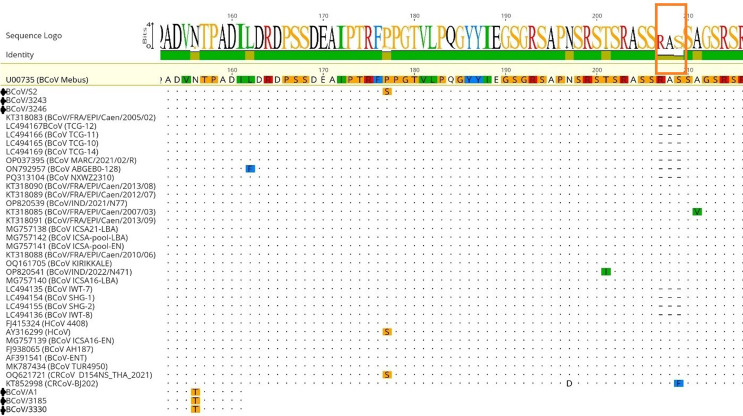
Amino acid sequence variations detected in the N gene region of the isolates

Analysis of the N gene amino acid sequences showed that BCoV/3243 and BCoV/3246 from August possess an identical three–amino acid deletion in the RS/SR domain, which was absent in BCoV/S2 from January. For BCoV/3185, BCoV/3330, and BCoV/A1, which were also obtained during the August outbreak, sequencing data could not be obtained due to the failure to generate first-round PCR products; therefore, the presence of the deletion in these viruses could not be assessed. However, sequence alignment of these samples revealed an N156T amino acid substitution (Fig. 2).

### S gene sequences and phylogenetic analysis

All samples that tested positive for BCoV in the N gene RT-PCR were tested for amplification of the S gene region using nine overlapping primer pairs (Supplementary Table 1), aiming to obtain the entire 4112 bp S gene sequence. The partial S gene sequences were successfully obtained from two samples, BCoV/S2 from January and BCoV/3246 from August, respectively (Table 1). For BCoV/S2, 80 amino acids could not be obtained within the region amplified by the SD primers, while for the 3246 sample, 110 amino acids were missing in the region targeted by the SG primers. As a result of the phylogenetic analysis conducted with these two samples, they clustered within the GIb subgroup together with previously reported European BCoV strains (Fig. 1b).

The S gene sequences of these strains (BCoV/S2 from January and BCoV/3246 from August) showed 98.55% nucleotide and 98.72% amino acid similarity to each other. Amino acid substitutions in the S gene sequences of mentioned strains were comprehensively analyzed using the Mebus strain as a reference and related sequences from the phylogenetic tree (Fig. 1b), revealing multiple amino acid changes across several regions (Supplementary Fig. S1). Analysis of the S1 domain (amino acids 438–599), which plays a critical role in viral tropism and antigenicity, revealed several amino acid substitutions common to both strains: (V465A, H470D, S484T, N509T, N531D, S543A, and Y571H). Strain-specific substitutions were also detected, including D492G and S510T in BCoV/3246, and N499S, P501F, and H525Y in BCoV/S2 (Table 2).

**Table 2:** Amino acid sequence variations detected in the S gene region (S1 domain) of the strains

	4	4	4	4	4	4	4	4	4	4	4	4	4	4	4	5	5	5	5	5	5	5	5	5	5	5	5	5	5	5	5	5	5	5	5	5	5	5	5
	3	4	5	5	5	6	6	6	6	7	7	8	8	9	9	0	0	0	0	0	0	0	1	1	2	2	2	3	3	3	4	4	4	4	4	4	5	5	7
	9	7	0	5	8	1	4	5	7	0	1	2	4	2	9	0	1	3	4	5	6	9	0	4	3	5	7	1	3	9	0	3	4	5	6	7	3	9	2
U00735 BCoV/Mebus	**S**	**T**	**R**	**T**	**F**	**K**	**P**	**V**	**V**	**H**	**H**	**A**	**S**	**D**	**N**	**G**	**P**	**I**	**D**	**A**	**G**	**N**	**S**	**T**	**T**	**H**	**A**	**N**	**L**	**I**	**T**	**S**	**T**	**G**	**P**	**Y**	**T**	**I**	**Y**
**PV975818 BCoV/3246**	.	.	.	.	.	.	.	A	.	D	.	.	T	G	.	.	.	.	.	.	.	T	T	.	.	.	.	D	.	.	.	A	.	.	.	.	.	.	H
**PV975817 BCoV/S2**	.	.	.	.	.	.	.	A	.	D	.	.	T	.	S	.	F	.	.	.	.	T	.	.	.	Y	.	D	.	.	.	A	.	.	.	.	.	.	H
MK989620 BCoV/4950	.	.	.	.	.	.	.	A	.	D	.	.	T	.	S	.	S	.	.	.	.	T	.	.	.	Y	.	D	.	.	.	A	.	.	.	.	.	.	H
MK989621 BCoV/4945	.	.	.	.	.	.	.	A	.	D	.	.	T	.	S	.	S	.	.	.	.	T	.	.	.	Y	.	D	.	.	.	A	.	.	.	.	.	.	H
MK989624 BCoV/S8	.	.	.	.	.	.	.	A	.	D	Y	.	T	.	S	.	S	.	.	.	.	.	.	.	.	Y	.	D	.	.	.	A	.	.	.	.	.	.	H
KT318114 BCoV/FRA/EPI/Caen/2008/04	.	.	.	.	S	.	.	A	.	D	.	.	T	.	S	.	S	.	.	.	.	T	.	.	.	Y	.	D	.	.	.	A	.	.	.	.	.	.	H
KT318119 BCoV/FRA/EPI/Caen/2013/09	.	.	.	.	.	.	.	A	.	D	.	.	T	.	S	.	F	.	.	.	.	.	.	.	.	Y	.	D	.	.	.	A	.	.	.	.	.	.	H
KT318112 BCoV/FRA/EPI/Caen/2005/02	.	.	.	.	S	.	.	A	.	D	.	.	T	.	S	.	S	.	.	.	.	.	.	.	.	Y	.	D	.	.	K	A	.	.	.	.	.	.	H
MK989622 BCoV/U1	.	.	.	.	S	.	.	A	.	D	.	.	T	.	S	.	S	.	.	.	.	T	.	.	.	Y	.	D	.	T	.	A	.	.	.	.	.	.	H
LC494165 BCoV/TCG-10	.	.	.	.	S	.	.	A	.	D	.	.	T	.	S	.	S	.	.	.	.	.	T	.	.	.	.	D	.	.	.	A	.	.	.	H	A	.	H
LC494167 BCoV/TCG-12	.	.	.	.	S	.	.	A	.	D	.	.	T	.	S	.	S	.	.	.	.	.	T	.	.	.	.	D	.	.	.	A	.	.	.	H	A	.	H
LC494166 BCoV/TCG-11	.	.	.	.	S	.	.	A	.	D	.	.	T	.	S	.	S	.	.	.	.	.	T	.	.	.	.	D	.	.	.	A	.	.	.	H	A	.	H
LC494169 BCoV/TCG-14	.	.	.	.	S	.	.	A	.	D	.	.	T	.	S	.	S	.	.	.	.	.	T	.	.	.	.	D	.	.	.	A	.	.	.	H	A	.	H
OP037395 BCoV/ MARC/2021/02/R	.	.	.	.	S	.	.	A	.	D	.	.	T	G	.	.	.	.	.	.	.	T	T	.	.	Y	.	D	.	.	.	A	.	.	S	.	.	.	H
ON792957 BCoV/ ABGEB0-128	.	.	.	.	.	.	.	A	.	D	.	.	T	.	S	.	S	.	.	.	.	T	.	.	.	Y	.	D	.	.	.	A	S	.	.	.	.	.	H
PQ313104 BCoV/ NXWZ2310	.	.	.	.	S	.	H	A	A	D	.	.	T	.	S	.	.	.	.	.	.	T	.	I	.	.	.	D	.	.	.	.	.	.	S	.	.	.	H
LC494135 BCoV/IWT-7	.	.	.	.	S	.	.	A	.	D	.	.	T	.	S	.	S	.	.	.	.	.	T	.	.	.	.	D	.	.	.	A	.	.	.	H	.	.	H
LC494154 BCoV/SHG-1	.	.	.	.	S	.	.	A	.	D	.	.	T	.	S	.	S	.	.	.	.	.	T	.	.	.	.	D	.	.	.	A	.	.	.	H	.	.	H
LC494155 BCoV/SHG-2	.	.	.	.	S	.	.	A	.	D	.	.	T	.	S	.	S	.	.	.	.	.	T	.	.	.	.	D	.	.	.	A	.	.	.	H	.	.	H
LC494136 BCoV/IWT-8	.	.	.	.	S	.	.	A	.	D	.	.	T	.	S	.	S	.	.	.	.	.	T	.	.	.	.	D	.	.	.	A	.	.	.	H	.	.	H
OP820541 BCoV/IND/2022/N471	.	.	.	.	S	.	.	?	A	D	.	.	T	.	.	.	S	.	.	.	.	T	.	.	.	Y	.	D	.	.	.	A	.	.	.	.	.	.	H
PP156987 BCoV/ S129	.	.	.	.	S	.	.	A	.	D	.	.	T	.	.	.	.	.	.	.	.	T	T	.	.	.	.	D	.	.	.	A	.	.	.	.	.	.	H
PP156982 BCoV/ S53 Albania/1-5671	.	.	.	.	S	.	.	A	.	D	.	.	T	.	S	.	F	.	.	.	.	T	.	.	.	Y	.	D	.	.	.	N	.	.	.	.	.	.	H
PP156981 BCoV/S52 Albania/1-5709	.	.	.	.	.	.	.	A	.	D	.	.	T	.	S	.	S	.	.	.	.	.	.	.	.	Y	.	D	M	.	.	A	.	.	.	.	.	.	H
PP156977 BCoV/S3 Albania/1-5715	.	.	K	.	S	.	.	A	.	D	Y	.	T	.	S	.	S	.	.	.	.	T	.	.	.	Y	.	D	.	.	.	A	.	.	.	.	.	.	H
MK989623 BCoV/T5	.	.	.	.	S	.	.	A	.	D	.	.	T	.	S	.	S	.	.	.	.	T	.	.	.	Y	.	D	.	.	.	A	.	.	.	.	.	.	H
AY935637 BCoV/KWD1	.	.	.	.	S	.	.	A	.	D	.	.	T	.	S	.	S	.	.	.	.	.	T	.	.	.	.	D	.	.	.	A	.	.	.	.	.	.	.
AY935638 BCoV/KWD2	.	.	.	.	S	.	.	A	.	D	.	.	T	.	S	.	S	.	.	.	.	.	T	.	.	.	.	D	.	.	.	A	.	.	.	.	.	.	.
AY935644 BCoV/KWD8	.	.	.	.	S	.	.	A	.	D	.	.	T	.	.	.	S	.	.	.	.	.	T	.	.	.	.	D	.	.	.	A	.	.	.	.	.	.	.

## Discussion

Bovine coronavirus (BCoV) is transmitted via feces and respiratory secretions, consistent with the organ systems affected by infection. Its efficient transmission and persistence in cattle populations lead to recurrent outbreaks, particularly in intensive production systems. Therefore, molecular analyses are essential for accurate pathogen identification and understanding BCoV epidemiology.

A total of 42 nasal swab and 10 fecal samples were collected from a cattle farm where respiratory outbreak was recorded during three distinct periods (January, May, and August) and enteric infections were observed in newborn calves only during the August outbreak. Of nasal swab samples, 12 tested positive (12/42; 28.57%). The positivity rates among sampled animals for the individual sampling periods were 6.7% (1/15), 33.3% (4/12), and 46.7% (7/15), respectively (Table 1).

BCoV was detected in calves presenting respiratory clinical signs during all three outbreak periods. The absence of other major respiratory viruses screened in the sampled animals suggests that BCoV was likely the primary agent responsible for the respiratory outbreaks in this farm.

Furthermore, BCoV was detected in one of the fecal samples collected during the August outbreak (1/10; 10%). Of course, this rate represents only the cases detected among the sampled animals, not the general incidence of diarrhea cases. It should be noted that bovine rotavirus was also detected in some samples (unpublished data). The detection of BCoV –and also bovine rotavirus- despite routine maternal vaccination may reflect incomplete passive protection in calves or the circulation of genetically diverse field strains.

Genetic characterization of viruses detected during outbreak events can provide useful molecular information on circulating strains and their genetic diversity. Therefore, this study focused on the molecular characterization of the N gene and, in particular, the S gene regions of BCoV detected in clinical samples collected during three distinct outbreak periods.

In this study, as in many previously reported studies, the N gene was targeted for viral detection because of its favorable diagnostic sensitivity (Lojkić et al. 2015; Mira Fernandes et al. 2018; Saif 2010; Li et al. 2024). Among the BCoV-positive samples, no sequence representing the May sampling period could be obtained. This may be related to low viral RNA concentration in these samples, which resulted in faint amplification products that were not suitable for reliable sequencing analysis. However, sequence data (*n* = 6) were successfully generated from first- and/or second-round RT-PCR products of samples from the other sampling periods: January and August. Owing to the longer sequence length, the phylogenetic tree was built using first-round RT-PCR sequences (Fig. 1a), which revealed that BCoV/S2 is located on a different branch than the one containing BCoV/3243 and BCoV/3246.

Based on the amino acid alignment of first-round N gene sequences from three strains detected in nasal samples, a distinct three–amino acid deletion (“RAS”) in the RS/SR domain was identified in two strains collected in August (BCoV/3243 and BCoV/3246) but was absent in the strain BCoV/S2 from January (Fig. 2). This deletion could not be evaluated in the remaining three samples collected in August (BCoV/3330 and BCoV/3185 from nasal samples, and BCoV/A1 from a fecal sample) due to unsuccessful amplification of the corresponding region. The aforementioned three-amino-acid deletion was observed in 12 BCoV isolates (MARC/2021/02/R, FRA/EPI/Caen/2005/02, SHG-1, SHG-2, IWT-7, IWT-8, TCG-10, TCG-11, TCG-12, TCG-14, ABGEB0-128, and NXWZ2310 strains) available in the NCBI database, which originated from either enteric or respiratory samples (Fig. 2) (Suzuki et al. 2020; Li et al. 2024; Qi et al. 2025). Of the isolates carrying this deletion, the NXWZ2310 strain has been associated with severe respiratory and enteric diseases (Li et al. 2024). It is noteworthy that the mentioned deletion was identified in strains from two nasal samples collected during a period associated with severe respiratory and diarrheal signs in August; however, a direct causal relationship cannot be established based on the current dataset. Unfortunately, the first-round N gene sequence of BCoV (BCoV/A1) detected in a diarrheic calf during the same period could not be obtained, preventing further evaluation. Nevertheless, the current findings suggest that this genetic feature is not limited to a specific clinical presentation. Another notable observation was the N156T substitution, which was identified in strains collected in August (BCoV/A1 from stool samples and BCoV/3185 and BCoV/3330 from nasal samples). This substitution was detected neither in the other samples from August (BCoV/3243 and BCoV/3246) in which the RAS deletion was present nor in the strain BCoV/S2 from January (Fig. 2). While this may indicate the concurrent circulation of different viral variants, it remains unclear whether it reflects viral evolution within the farm or the introduction of a distinct strain.

Several studies have indicated that certain amino acid changes in the S gene can affect receptor affinity and alter biological characteristics, such as tissue tropism and pathogenicity (Schultze et al. 1991; Hulswit et al. 2016). In recent studies, based on S gene phylogenetic analysis, BCoV strains have been mainly classified into two major groups: GI and GII. The GIa subgroup contains strains from Asia, America, Europe, and the original Mebus strain, whereas the GIb subgroup consists mainly of European strains. Most American and Asian strains cluster in the GII group, where Korean strains form a distinct GIIa subgroup, and strains from America, Japan, Vietnam, and China group into GIIb (Zhu et al. 2022). In this study, the S gene regions of two strains (BCoV/S2 and BCoV/3246) from different sampling periods, January and August, were analyzed, and they clustered within the GIb subgroup (Fig. 1b).

The identification of different amino acid substitutions in the S gene region, along with those observed in the N gene region, further supports the genetic diversity of the circulating BCoVs. Briefly, in the S1 subunit, the K179R amino acid substitution detected only in BCoV/3246 was also observed in isolates from Japan (LC494166, LC494167) and several Korean isolates (AY935637, AY935638, AY935639, AY935640, and AY935644) belonging to a different phylogenetic group (GII) (Supplementary Fig. S1). Upon examining the S1 domain (amino acids 438–599), which plays an important role in viral tropism and antigenicity, several amino acid substitutions were also identified in our samples (Table 2). The S510T substitution, observed exclusively in BCoV/3246, is particularly noteworthy, as it is also commonly present in most GII group strains, including those from Korea and Japan. Schultze et al. (1991) reported that amino acid residues within the S1 domain, particularly at positions 510–530, are crucial for binding to 9-O-acetylated sialic acid, which is essential for effective infection of the respiratory epithelium. The S510T substitution has been linked to changes in receptor affinity and altered hemagglutination characteristics (Schultze et al. 1991; Hulswit et al. 2016). It has been suggested that S510T and N531D changes may be markers of respiratory tropism (Hasoksuz et al. 2005). However, not all studies have found consistent associations between these substitutions and tissue tropism. Their detection in some enteric isolates (Jeong et al. 2005; Sevinc-Temizkan and Alkan 2021; Suzuki et al. 2020) suggests that the relationship between these mutations and tissue tropism remains inconclusive. Our two strains also differed from each other with respect to substitutions such as D492G, N499S, P501F, and H525Y, which have previously been reported in strains from Türkiye and other countries (Table 2). Considering that one strain (BCoV/S2) was detected in January and the other (BCoV/3246) in August, and that the August strain harbored a three-amino-acid deletion in the RS/SR domain of the N gene, this observation may reflect the presence of genetically distinct strains circulating within the farm over time. In contrast, the A528V substitution described by Yoo and Deregt (2001) in antigenic domain II—which was shown to confer resistance to neutralizing monoclonal antibodies under in vitro selection pressure—was not detected in either strain (Table 2).

A limitation of this study is the restricted number of successfully obtained sequences across all sampling periods, particularly the inability to generate sequence data from the May outbreak and from some BCoV-positive samples in August. This limited sequencing coverage may have reduced the ability to fully capture the within-farm genetic diversity of circulating strains and to comprehensively assess possible co-circulation of multiple variants. In addition, the study was conducted on a single farm, which limits the generalizability of the findings to broader cattle populations. Finally, the lack of complete genome data and reliance on partial N and S gene sequences constrain the deeper resolution of evolutionary relationships and functional interpretations.

In conclusion, this study, based on partial N and S gene sequence analysis, demonstrates that genetically distinct BCoV strains can circulate over time within a large-scale cattle farm, although it does not address their potential impact on clinical severity, viral load, or other relevant factors. Future studies, including additional genomic regions, whole-genome sequencing, and functional analyses, will help to better understand the epidemiology and biological characteristics of BCoVs.

## Supplementary Information

Below is the link to the electronic supplementary material.

Supplementary Material 1: Alignment of amino acid sequences showing variations in the spike (S) gene region among bovine coronavirus strains

Supplementary Material 2: Primers used for amplification of the N gene and full‑length S gene (including S1 and S2 subunits).

## Data Availability

All data generated or analyzed during this study are included in this published article.
