# Supplementary material for: Genetic diversity of bovine coronaviruses from recurrent outbreaks on a large-scale cattle farm
Source: Trop Anim Health Prod. 2026 Jun 4;58(5):315. doi: 10.1007/s11250-026-05112-8 (PMC13236805; doi:10.1007/s11250-026-05112-8)
Supplement: Supplementary file 1 — Supplementary Material 1: Alignment of amino acid sequences showing variations in the spike (S) gene region among bovine coronavirus strains [file 11250_2026_5112_MOESM1_ESM.pdf]

Article Title:
Genetic Diversity of Bovine Coronaviruses from Recurrent Outbreaks on a Large-Scale Cattle Farm

Journal Name:
Tropical Animal Health and Production

Author Names and Affiliations:

Selda Duran-Yelken[1*], Ilke Karayel-Hacioglu[2,3], Zelfinaz Aydin[4], Feray Alkan[2,3]

[1]Department of Virology, Faculty of Veterinary Medicine, Kastamonu University, Kastamonu, Türkiye

[2]Department of Virology, Faculty of Veterinary Medicine, Ankara University, Ankara, Türkiye

[3]Graduate School of Health Sciences, Ankara University, Ankara, Türkiye

[4]Faculty of Veterinary Medicine, Kastamonu University, Kastamonu, Türkiye

***Corresponding author:** Selda Duran-Yelken, syelken@kastamonu.edu.tr

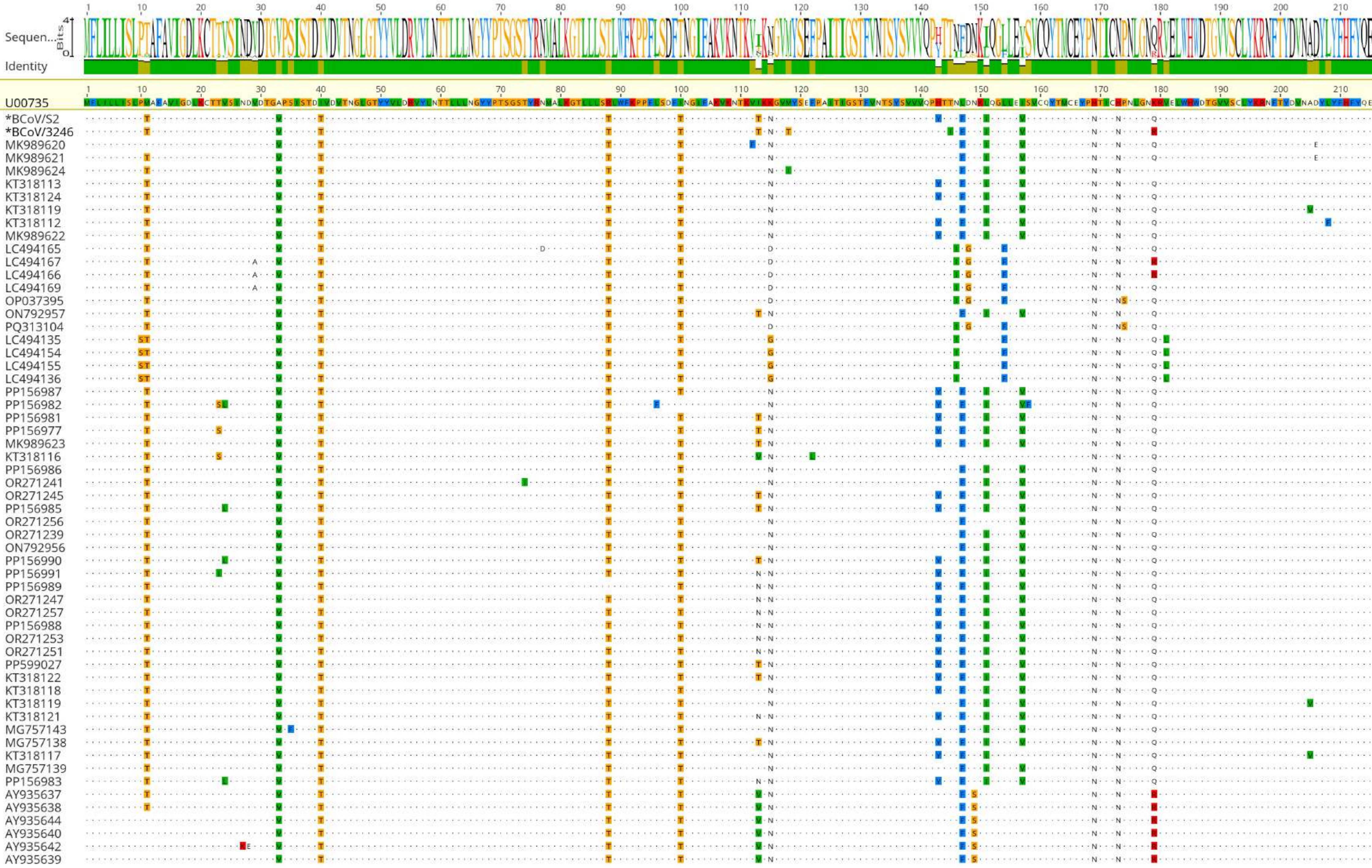
Sequen...
Bits
Identity
U00735
*BCoV/S2
*BCoV/3246
MK989620
MK989621
MK989624
KT318113
KT318124
KT318119
KT318112
MK989622
LC494165
LC494167
LC494166
LC494169
OP037395
ON792957
PQ313104
LC494135
LC494154
LC494155
LC494136
PP156987
PP156982
PP156981
PP156977
MK989623
KT318116
PP156986
OR271241
OR271245
PP156985
OR271256
OR271239
ON792956
PP156990
PP156991
PP156989
OR271247
OR271257
PP156988
OR271253
OR271251
PP599027
KT318122
KT318118
KT318119
KT318121
MG757143
MG757138
KT318117
MG757139
PP156983
AY935637
AY935638
AY935644
AY935640
AY935642
AY935639

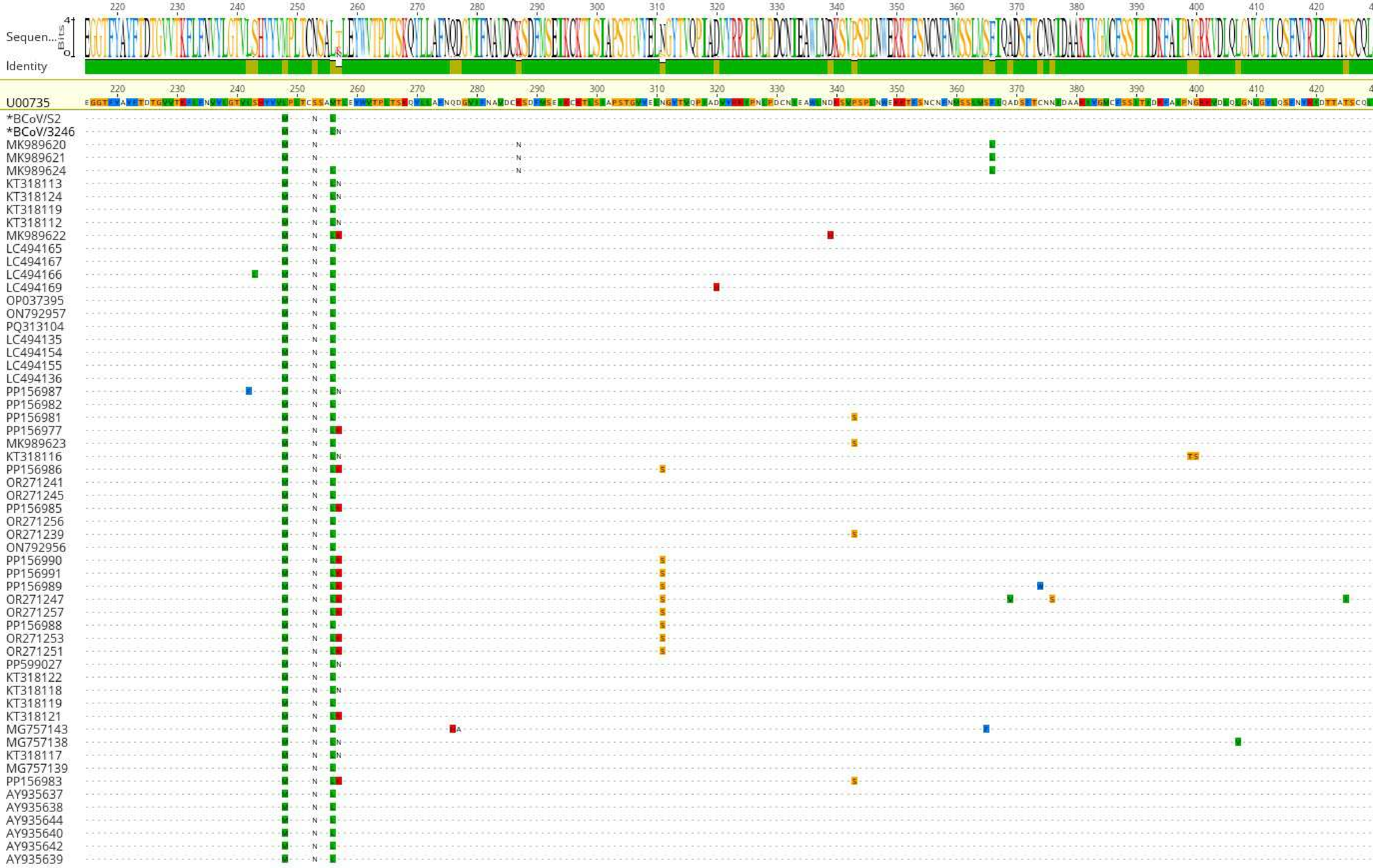

Sequen...
Bits
Identity
220
230
240
250
260
270
280
290
300
310
320
330
340
350
360
370
380
390
400
410
420
U00735
*BCoV/S2
*BCoV/3246
MK989620
MK989621
MK989624
KT318113
KT318124
KT318119
KT318112
MK989622
LC494165
LC494167
LC494166
LC494169
OP037395
ON792957
PQ313104
LC494135
LC494154
LC494155
LC494136
PP156987
PP156982
PP156981
PP156977
MK989623
KT318116
PP156986
OR271241
OR271245
PP156985
OR271256
OR271239
ON792956
PP156990
PP156991
PP156989
OR271247
OR271257
PP156988
OR271253
OR271251
PP599027
KT318122
KT318118
KT318119
KT318121
MG757143
MG757138
KT318117
MG757139
PP156983
AY935637
AY935638
AY935644
AY935640
AY935642
AY935639

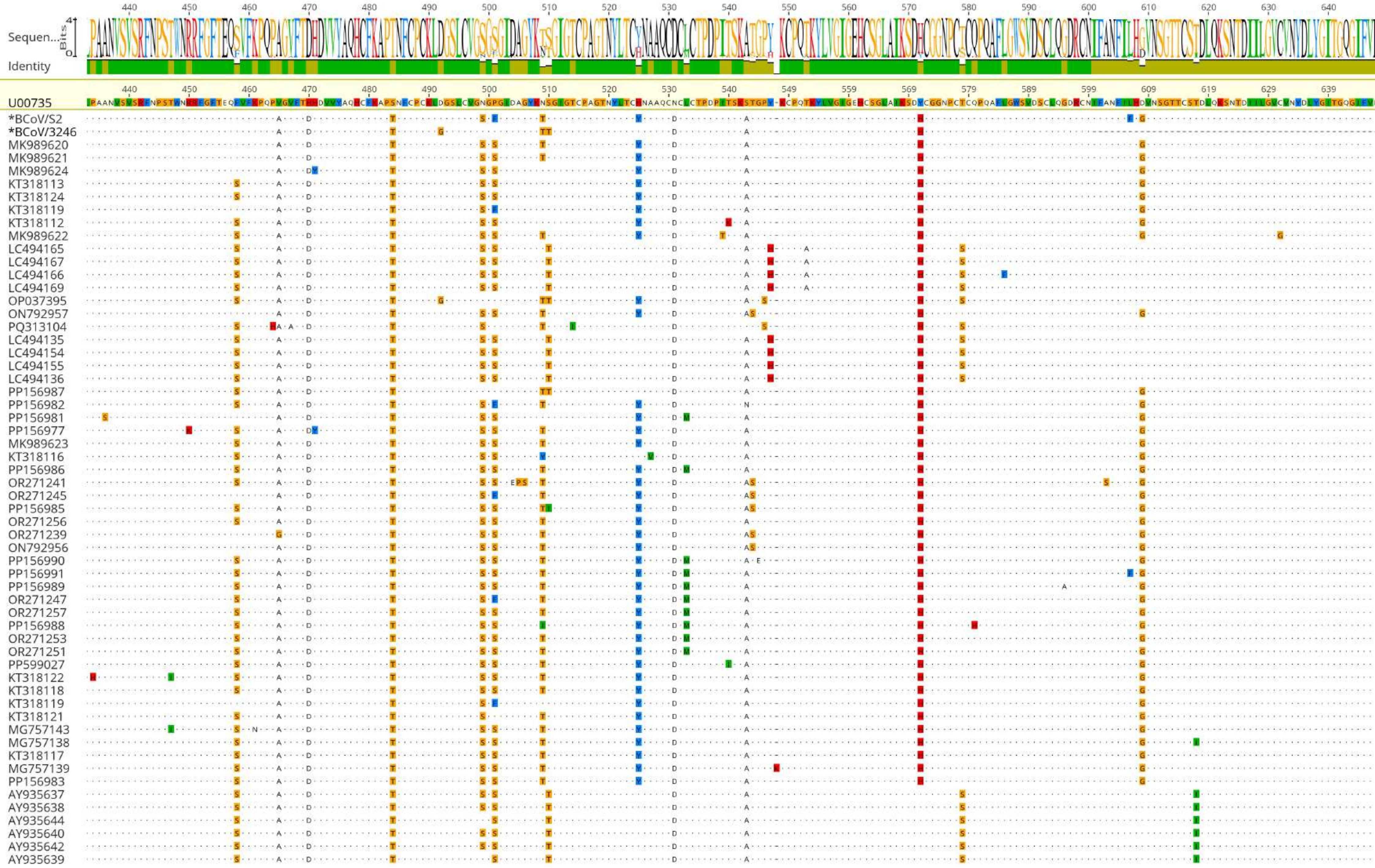

Sequen...
Bits
Identity
U00735
*BCoV/S2
*BCoV/3246
MK989620
MK989621
MK989624
KT318113
KT318124
KT318119
KT318112
MK989622
LC494165
LC494167
LC494166
LC494169
OP037395
ON792957
PQ313104
LC494135
LC494154
LC494155
LC494136
PP156987
PP156982
PP156981
PP156977
MK989623
KT318116
PP156986
OR271241
OR271245
PP156985
OR271256
OR271239
ON792956
PP156990
PP156991
PP156989
OR271247
OR271257
PP156988
OR271253
OR271251
PP599027
KT318122
KT318118
KT318119
KT318121
MG757143
MG757138
KT318117
MG757139
PP156983
AY935637
AY935638
AY935644
AY935640
AY935642
AY935639

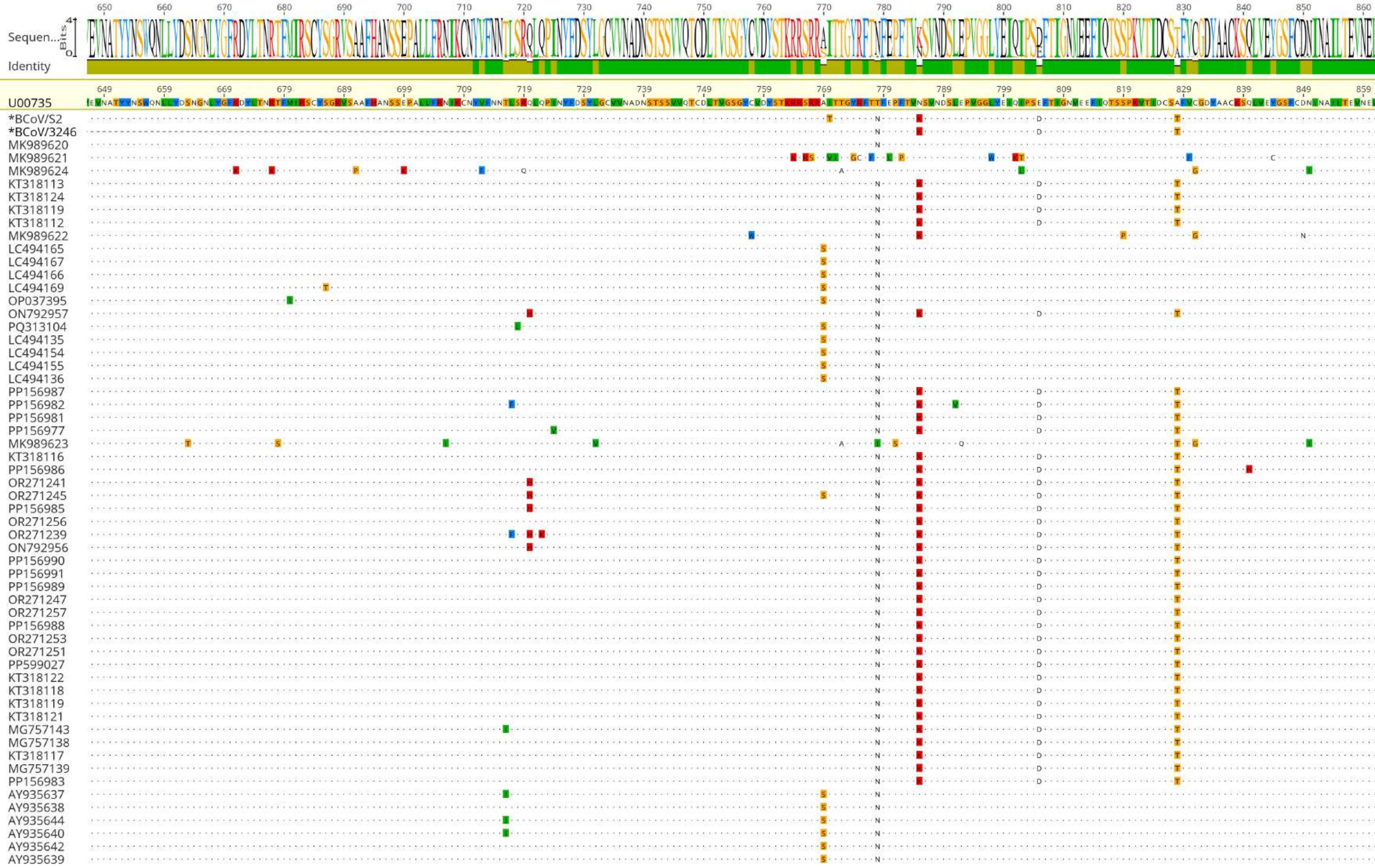
Sequen...
Bits
Identity
U00735
*BCoV/S2
*BCoV/3246
MK989620
MK989621
MK989624
KT318113
KT318124
KT318119
KT318112
MK989622
LC494165
LC494167
LC494166
LC494169
OP037395
ON792957
PQ313104
LC494135
LC494154
LC494155
LC494136
PP156987
PP156982
PP156981
PP156977
MK989623
KT318116
PP156986
OR271241
OR271245
PP156985
OR271256
OR271239
ON792956
PP156990
PP156991
PP156989
OR271247
OR271257
PP156988
OR271253
OR271251
PP599027
KT318122
KT318118
KT318119
KT318121
MG757143
MG757138
KT318117
MG757139
PP156983
AY935637
AY935638
AY935644
AY935640
AY935642
AY935639

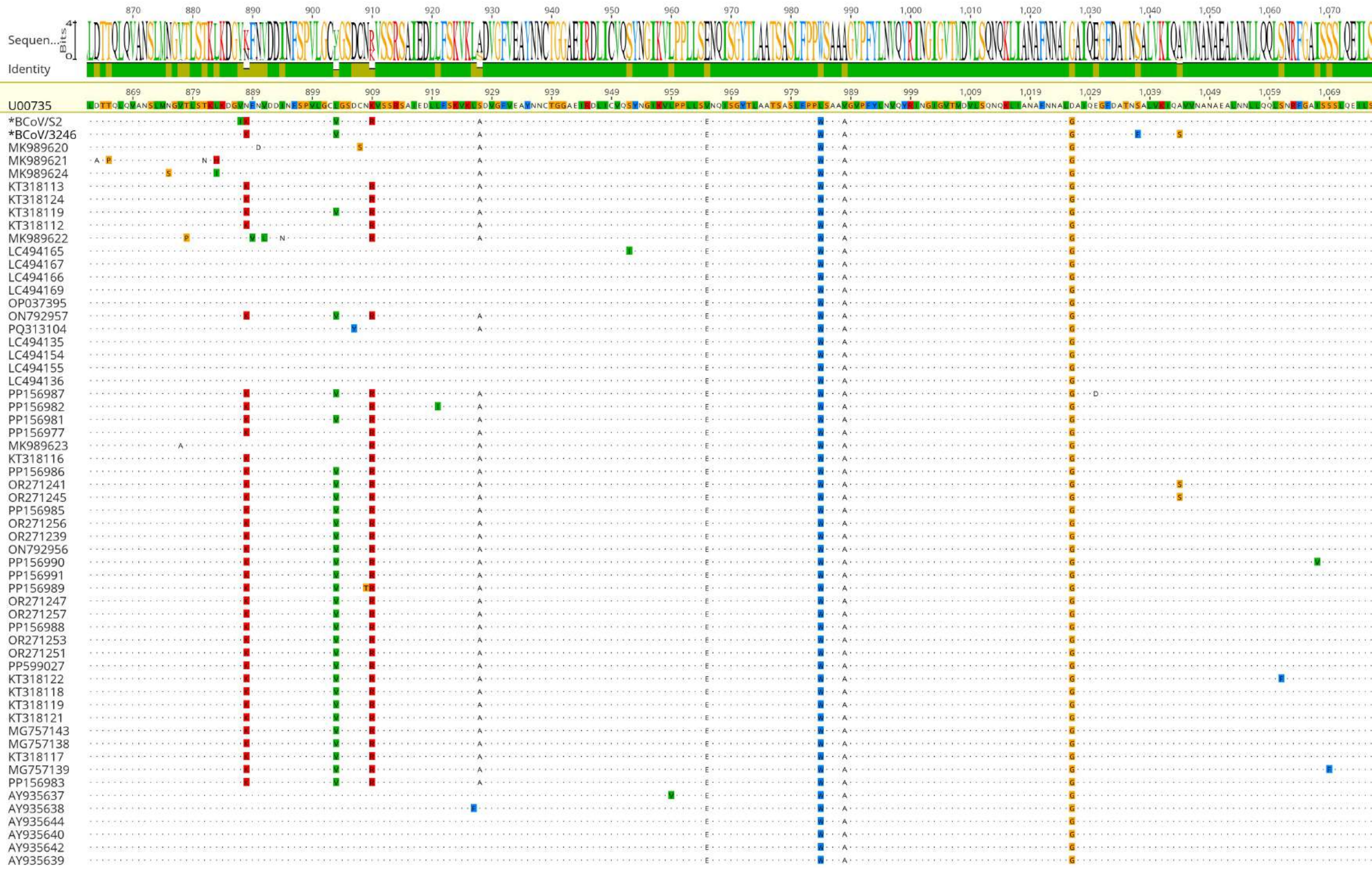
Sequen...
Bits
Identity
U00735
*BCoV/S2
*BCoV/3246
MK989620
MK989621
MK989624
KT318113
KT318124
KT318119
KT318112
MK989622
LC494165
LC494167
LC494166
LC494169
OP037395
ON792957
PQ313104
LC494135
LC494154
LC494155
LC494136
PP156987
PP156982
PP156981
PP156977
MK989623
KT318116
PP156986
OR271241
OR271245
PP156985
OR271256
OR271239
ON792956
PP156990
PP156991
PP156989
OR271247
OR271257
PP156988
OR271253
OR271251
PP599027
KT318122
KT318118
KT318119
KT318121
MG757143
MG757138
KT318117
MG757139
PP156983
AY935637
AY935638
AY935644
AY935640
AY935642
AY935639

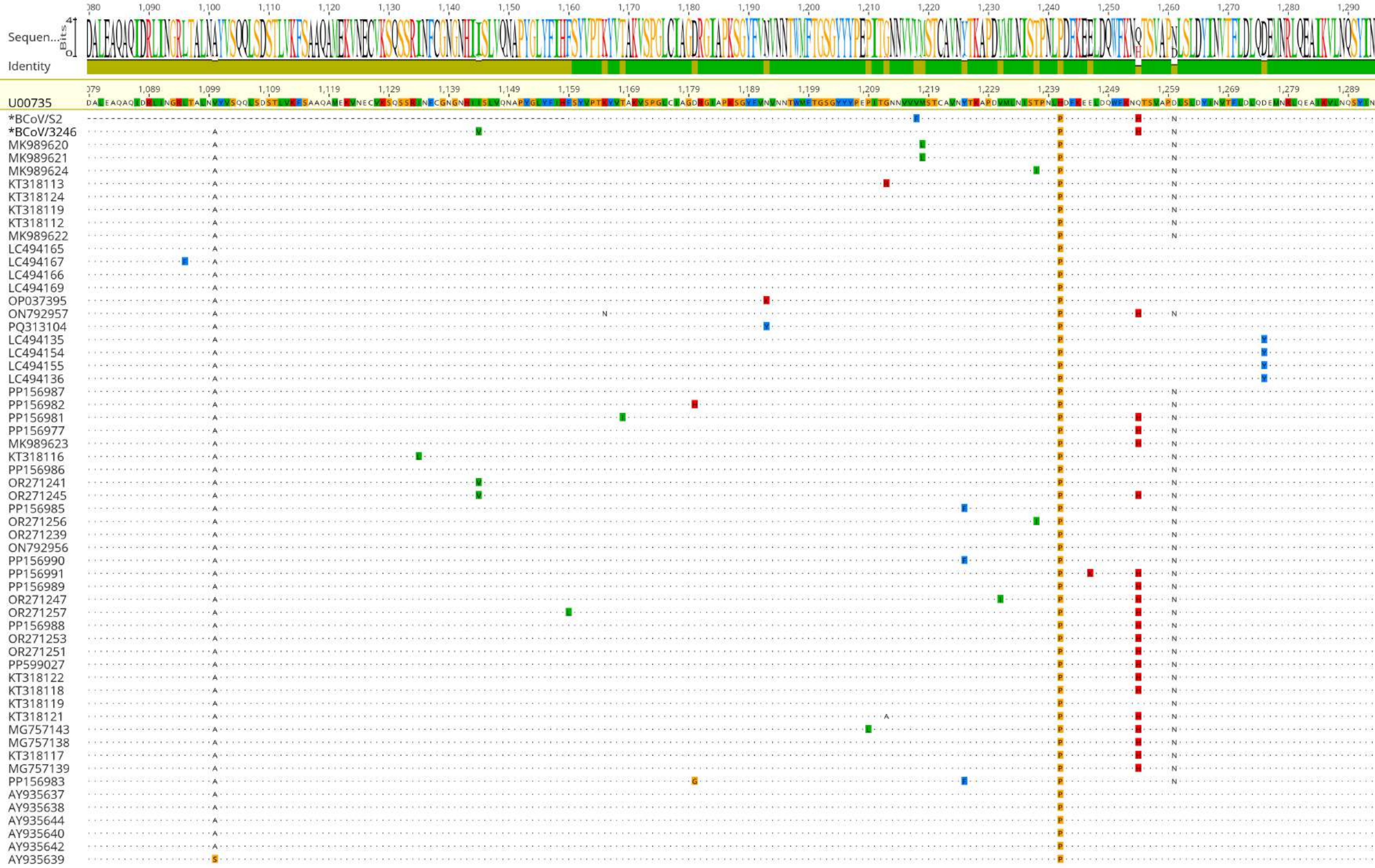

Sequen...
Bits
Identity
U00735
*BCoV/S2
*BCoV/3246
MK989620
MK989621
MK989624
KT318113
KT318124
KT318119
KT318112
MK989622
LC494165
LC494167
LC494166
LC494169
OP037395
ON792957
PQ313104
LC494135
LC494154
LC494155
LC494136
PP156987
PP156982
PP156981
PP156977
MK989623
KT318116
PP156986
OR271241
OR271245
PP156985
OR271256
OR271239
ON792956
PP156990
PP156991
PP156989
OR271247
OR271257
PP156988
OR271253
OR271251
PP599027
KT318122
KT318118
KT318119
KT318121
MG757143
MG757138
KT318117
MG757139
PP156983
AY935637
AY935638
AY935644
AY935640
AY935642
AY935639

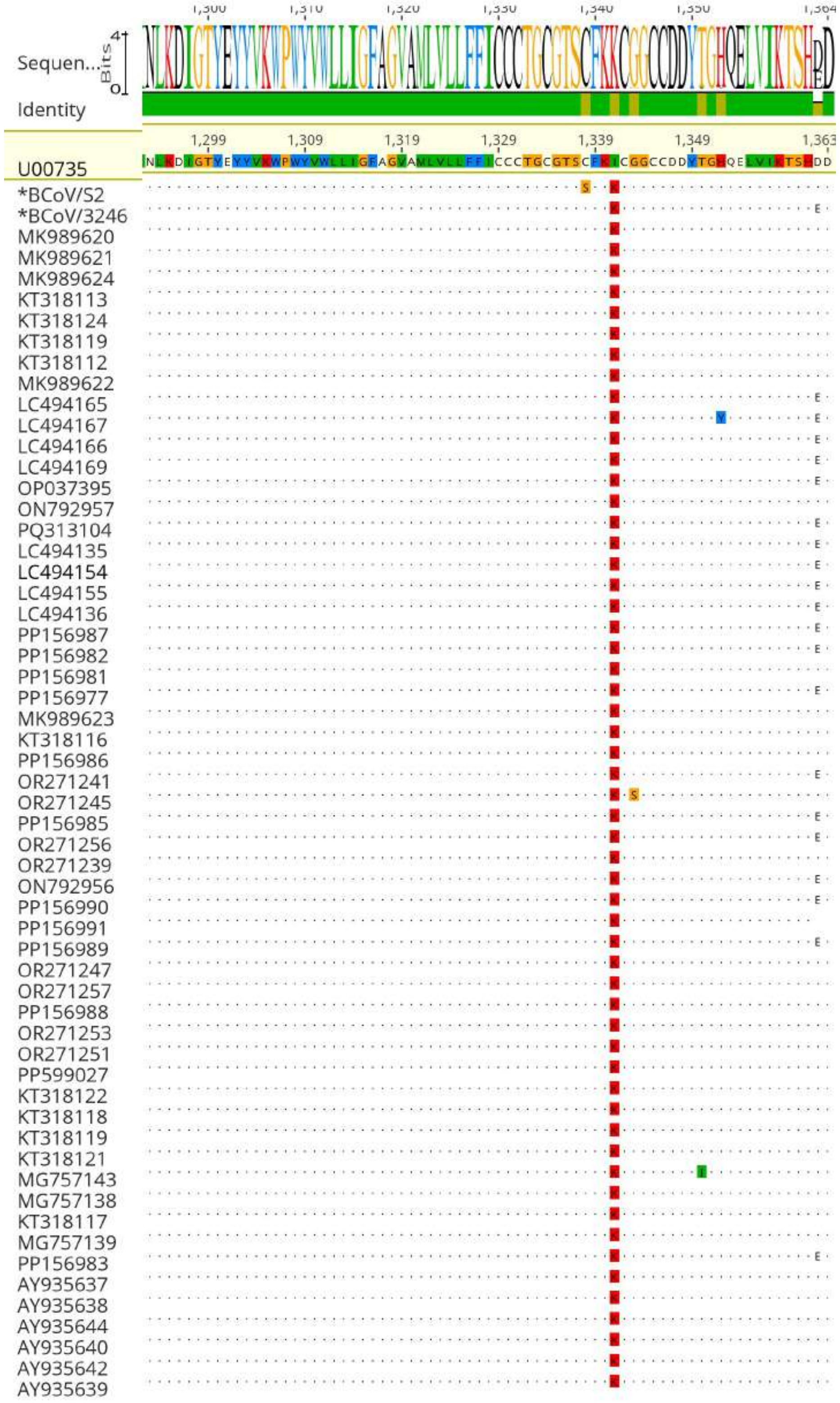

Supplementary Fig. 1 Alignment of amino acid sequences showing variations in the spike (S) gene region among bovine coronavirus strains
