# Supplementary material for: Genetic diversity of bovine coronaviruses from recurrent outbreaks on a large-scale cattle farm
Source: Trop Anim Health Prod. 2026 Jun 4;58(5):315. doi: 10.1007/s11250-026-05112-8 (PMC13236805; doi:10.1007/s11250-026-05112-8)
Supplement: Supplementary file 2 — Supplementary Material 2: Primers used for amplification of the N gene and full‑length S gene (including S1 and S2 subunits). [file 11250_2026_5112_MOESM2_ESM.pdf]

Article Title:

Genetic Diversity of Bovine Coronaviruses from Recurrent Outbreaks on a Large-Scale Cattle Farm

Journal Name:

Tropical Animal Health and Production

Author Names and Affiliations:

Selda Duran-Yelken[1*], Ilke Karayel-Hacioglu[2,3], Zelfinaz Aydin[4], Feray Alkan[2,3]

[1]Department of Virology, Faculty of Veterinary Medicine, Kastamonu University, Kastamonu, Türkiye

[2]Department of Virology, Faculty of Veterinary Medicine, Ankara University, Ankara, Türkiye

[3]Graduate School of Health Sciences, Ankara University, Ankara, Türkiye

[4]Faculty of Veterinary Medicine, Kastamonu University, Kastamonu, Türkiye

***Corresponding author:** Selda Duran-Yelken, syelken@kastamonu.edu.tr

Supplementary Table 1 Primers used for amplification of the N gene and full-length S gene (including S1 and S2 subunits).

Primer Name	Target Gene Region	Primer Sequence (5'→3')	Annealing (°C)	Location	Amplicon Size	References
BCoV N-F	N (1st round)	GCAATCCAGTAGTAGAGCGT	50°C	21-40	730 bp	Cho et al. 2001
BCoV N-R		CTTAGTGGCATCCTTGCCAA		731-750		
nBCoV N-F	N (2nd round)	GCCGATCAGTCCGACCAATG	58°C	79-98	407 bp	
nBCoV N-R		AGAATGTCAGCCGGGGTAG		467-485		
S-AF	S-1	ATGTTTTTGATACTTTTAATT	51°C	1-21	655bp	Hasoksuz et al. 2002
S-AR		AGTACCACCTTCTTGATAAA		654-635		
S-BF		ATGGCATTGGGATACAG	55°C	549-565	490 bp	
S-BR		TAATGGAGAGGGCACCGACTT		1039-1018		
S-CF		GGGTTACACCTCTCACTTCT	58°C	782-801	769bp	
S-CR		GCAGGACAAGTGCCTATACC		1550-1531		
S-DF		GTCCGTGTAAATTGGATGGG	55°C	1460-1479	827bp	
S-DR		TGTAGAGTAATCCACACAGT		2286-2267		
S-1F		GTTTCTGTTAGCAGGTTTAA		1329-1348	488 bp	Brandao et al. 2006
S-1R		ATATTACACCTATCCCCTTG		1797-1816		
S-EF	S-1 S-2	TTACAAAAATCAAACACAGACAT		1855-1877	877bp	Hasoksuz et al. 2002
S-ER		AAACTTTATTACAATCGCTTCC		2731-2710		
S-FF	S-2	TCAATTTTTCCCCTGTATTAGG		2680-2702	555 bp	Martinez et al. 2012
S-FR		CMAGTCTRGATAGAATTTCTTGTAA		3234-3209		
S-GF		GCTACCAATTCTGCTTTAGTTA		3099-3121	519 bp	
S-GR		GTAGTAATAACCACTACCAGTG		3617-3595		
S-HF		TTTAGCTATGTCCCTACTAAGTA	58°C	3475-3498	637 bp	
S-HR		CCAATAAATCAAAGACGAACTTA		4112-4089		
